# The role of conserved residues of chagasin in the inhibition of cysteine peptidases

**DOI:** 10.1016/j.febslet.2008.01.008

**Published:** 2008-02-20

**Authors:** Flavia C.G. dos Reis, Brian O. Smith, Camila C. Santos, Tatiana, F.R. Costa, Julio Scharfstein, Graham H. Coombs, Jeremy C. Mottram, Ana Paula C.A. Lima

**Affiliations:** a Instituto de Biofísica Carlos Chagas Filho, Universidade Federal do Rio de Janeiro, Bloco G, C.C.S., Cidade Universitária, Ilha do Fundão, Rio de Janeiro, 21949-900 RJ, Brazil; b Divisions of Biochemistry and Molecular Biology, Institute of Biomedical Life Sciences, University of Glasgow, Glasgow G12 8QQ, UK; c Institute of Biomedical Life Sciences, Wellcome Centre for Molecular Parasitology, University of Glasgow, Glasgow G12 8QQ, UK

**Keywords:** Z-Phe-Arg-MCA, carbobenzoxy-phenylalanyl-arginyl-7-amido-4-methylcoumarin, PBS, phosphate buffered saline, cruzain, recombinant cruzipain truncated at the C-terminal extension, DTT, dithiothreitol, EDTA, ethylenidiaminetetracetic acid disodium salt 2-hydrate, E-64, l-*trans*-epoxysuccinylleucylamido-(4-guanidino) butane, IPTG, isopropyl-β-d-thiogalactopyranoside, Chagasin, Cysteine peptidase, Inhibitor, Mutant, *Trypanosoma*

## Abstract

We have evaluated the roles of key amino acids to the action of the natural inhibitor chagasin of papain-family cysteine peptidases. A W93A substitution decreased inhibitor affinity for human cathepsin L 100-fold, while substitutions of T31 resulted in 10–100-fold increases in the *K*_*i*_ for cruzipain of *Trypanosoma cruzi*. A T31A/T32A double mutant had increased affinity for cathepsin L but not for cruzipain, while the T31-T32 deletion drastically affected inhibition of both human and parasite peptidases. These differential effects reflect the occurrence of direct interactions between chagasin and helix 8 of cathepsin L, interactions that do not occur with cruzipain.

## 1 Introduction

Chagasin is a natural inhibitor of Clan CA, Family C1 cysteine peptidases (CP), which was identified in the protozoan *Trypanosoma cruzi*, the causative agent of human Chagas’ disease [1,2]. It is a single 110-residue polypeptide chain with no sequence similarity to other known groups of CP inhibitors and potently inactivates papain-like enzymes through the formation of 1:1 tight–binding complexes. Chagasin-like inhibitors which occur in other protozoa and bacteria [3] have been designated as ICP (*I*nhibitor of *C*ysteine *P*eptidases), and together comprise the chagasin family (Clan XI – Family I42) [3,4].

Genetic evidence indicates that chagasin itself regulates the activity of cruzipain, the major CP activity of *T. cruzi*. This conclusion is based on the findings that *T. cruzi* lines that overexpress chagasin have an 80% decrease in active CPs, are less capable to differentiate and are less infective to mammalian cells in vitro [5].

The solution structures of chagasin and of *Leishmania mexicana* ICP determined by NMR showed that the molecule has an Ig-like fold and three exposed loop regions, which bear evolutionarily conserved residues, located at one end of the molecule [6,7]. These three key loops, L2 (residues 27–33), L4 (residues 59–69) and L6 (residues 91–100) contain, respectively: (i) the NPTTG motif; (ii) a conserved region bearing two hydrophobic residues followed by GXGG; and (iii) the RPW/F motif [6]. Studies of chemical shift perturbations upon chagasin contact with cruzipain identified residues in L2, L4 and L6 as candidates to comprise chagasin’s binding-site to target enzymes [6]. This hypothesis was reinforced by docking of a high resolution crystal structure of chagasin to the high-resolution structure of cruzain (truncated recombinant cruzipain) [8]. The recent determination of the chagasin–falcipain2 [9] and chagasin–cathepsin L [10] complex structures revealed that L2 is in close proximity to the peptidases’ catalytic triad and that the conserved residues T31 and T32 of chagasin mediate the formation of key hydrogen bonds with the peptidases, mainly through water molecules. In contrast, the conserved P30 seems to fulfill a structural role. The conserved residues within L6 appear to make mainly hydrophobic interactions with the peptidases, and could ultimately contribute to high affinity inhibition. These findings raised the question of the relative contributions of the conserved residues to chagasin’s function as an inhibitor, the question that the present study set out to address.

## 2 Materials and methods

### 2.1 Site-directed mutagenesis

*T. cruzi* (Dm28c) chagasin, was cloned into the pQE30 expression vector to produce a fusion with a 6X-Histidine tag. Mutants of chagasin were obtained by site-directed mutagenesis using specific oligonucleotides (Table 1). All plasmids were confirmed by DNA sequencing.

### 2.2 Expression and purification of recombinant chagasin variants

Overnight cultures of *Escherichia coli* M15/pRep4 containing the various chagasin plasmids were diluted 20-fold in fresh LB medium containing 50 μg/mL ampicillin and 25 μg/mL kanamicin and grown for 1 h at 37 °C followed by addition of 1 mM isopropyl-β-d-thiogalactopyranoside (IPTG) and incubation overnight at 16 °C. The cells were lysed by sonication in 50 mM Na_2_PO_4_, pH 8, 300 mM NaCl, 10 mM imidazole. The soluble fraction was collected by centrifugation at 10 000 × *g* and filtered. The sample was applied to a Metal Chelate Ni^+^ Column (BioCAD, BioSystems). The resin was washed with 50 mM Na_2_PO_4_, pH 7.9, 300 mM NaCl, 50 mM imidazole and the recombinant proteins were eluted in the same buffer supplemented with 500 mM imidazole. The eluted samples were further purified by HQ Ion Exchange in 50 mM Tris–HCl, pH 7, 5 mM ethylenidiaminetetracetic acid disodium salt 2-hydrate (EDTA), eluted in the same buffer over a 1 M NaCl gradient and the fractions were pooled. The purity of the samples was analysed by 14% SDS–PAGE, followed by Coomassie staining.

### 2.3 Heat stability

Wild type chagasin and the variants were diluted to 90 μg/ml in 20 mM phosphate buffer, 150 mM NaCl pH 7.2 (PBS) and incubated in a water bath at 70 °C for 20 min or kept on ice. The inhibitors were subsequently incubated at 2 μg/ml with 0.07 nM of papain in 50 mM Na_2_PO_4_, pH 6.5, 200 mM NaCl, 5 mM EDTA, 2.5 mM dithiothreitol (DTT) for 5 min at room temperature. Enzyme activity was subsequently monitored by the hydrolysis of 5 μM of carbobenzoxy-phenylalanyl-arginyl-7-amido-4-methylcoumarin (Z-Phe-Arg-MCA). Initial velocities were determined from linear regression on the substrate hydrolysis curves and used to calculate the percentage of inhibition in relation to controls kept on ice.

### 2.4 Enzyme inhibition assays

Concentrations of inhibitory chagasin were determined by titration with papain, which had been previously titrated with E-64 [11]. Different volumes of the recombinant chagasin and 100 nM papain solution and were incubated for 30 min at room temperature in RB buffer (50 mM Na_2_PO_4_, pH 6.5, 200 mM NaCl, 5 mM EDTA, 2.5 mM DTT). Aliquots of 5 μL of the incubation were diluted in 1 mL of the same buffer and residual enzyme activity was measured by the hydrolysis of 5 μM Z-Phe-Arg-MCA. Substrate hydrolysis was monitored in a F4500 Hitachi fluorimeter at 380 nm excitation, 440 nm emission and the initial velocities were calculated by linear regression. The determination of the equilibrium constants for dissociation (*K*_*i*_) were performed in RB buffer at 25 °C, as described for tight inhibitors [12]. The following CPs: cruzipain [13], papain (Sigma) and human cathepsin L (Calbiochem) were incubated with different chagasin concentrations for 15 min at room temperature and the residual velocities were measured by the hydrolysis of 5 μM Z-Phe-Arg-MCA. The *K*_*i*app_ values were determined from the slope of a plot of [*I*]_0_/1 − *a*, against 1/*a*, where *a* = *V*_*i*_/*V*_0_. *K*_*i*_ constants were calculated from *K*_*i*app_ values using the *K*_M_ values of 1.5 μM, 2.5 μM, 60 μM, for cruzipain, cathepsin L, and papain, respectively. The *K*_*i*_ values of some of the mutants with cathepsin L were further confirmed by the Dixon method in continuous assays using [*S*] ≪ *K*_M_.

### 2.5 Homology based modeling

Models of the chagasin–peptidase complexes were built using Modeller 9.1 [14] based on the coordinates of the chagasin–falcipain2 complex (9; PDB 2oul). For cruzain, PDB entries 1aim, 1me4 and 1me3 were aligned with falcipain 2 using Modeller’s structure based alignment algorithm to serve as templates for the complex.

## 3 Results and discussion

### 3.1 Production of chagasin variants

**Fig. 1:**
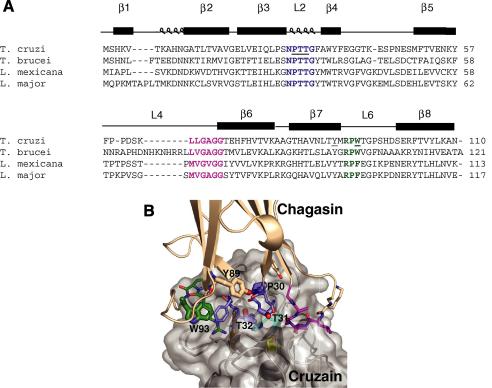
Chagasin residues targeted for mutagenesis. (A) Sequence alignment of chagasin (AJ299433), *L. major* ICP (AJ548878), *L. mexicana* ICP (AJ548776) and *T. brucei* ICP (AJ548777). The secondary structure is assigned on top and the conserved motifs are marked in bold. Conserved residues in loops L2, L4 and L6 are coloured blue, magenta, and green. The residues selected for site-directed mutagenesis are underlined. (B) Detail of the modeled interface between chagasin (beige) and cruzain (grey). Chagasin loops L2, L4 and L6 are coloured blue, magenta and green, respectively. Residues responsible for direct peptidase recognition and the peptidase’s catalytic triad are shown as sticks, and those mutated in this study are labeled and highlighted as fatter sticks.

We used the findings from the studies of chagasin docking to cruzipain and chemical shift perturbation as a base to guide the site-directed mutagenesis [6,8]. The solution structure of chagasin suggested that the spatial arrangement of Loop2 (L2) might be influenced by the conserved Y89 residue, which is present on the β7 strand in close proximity to L2, and protrudes towards S28 [6]. Thus we selected this residue and also residues P30, T31, T32, composing the conserved NPTTG motif (L2), and the conserved W93 (L6) as targets for amino acid replacement in chagasin variants (Fig. 1A). The relative position of the residues selected for mutagenesis at the inhibitor–peptidase interface is shown in Fig. 1B. The relative importance of the side chains of T31 and T32 was investigated by studying variants bearing substitutions to charged, apolar and hydrophobic residues. We also addressed the importance of the length of L2 by the generation of a mutant devoid of residues T31 and T32 (see Table 1). All variants were generated using similar methods and SDS–PAGE of the purified proteins under reducing conditions showed that all variants had mobilities similar to that of chagasin (data not shown).

**Fig. 2:**
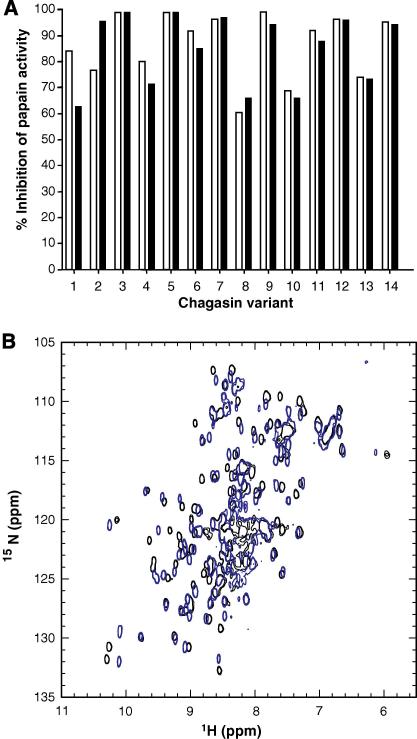
Thermo-stability properties of chagasin mutants. (A) Chagasin variants were submitted to high temperatures and subsequently tested for inhibitory activity to evaluate if the mutations introduced caused alterations in their folding state. The inhibitory activity of variants kept on ice was measured in parallel as a control. Chagasin variants were kept on ice (clear bars) or incubated at 70 °C for 20 min (dark bars). The samples were subsequently incubated with papain for 5 min and the residual activity was measured by addition of Z-Phe-Arg-MCA. The experiment was performed twice independently and the variation was below 10%. The graph represents one experiment. The percentage of inhibition was calculated considering the activity of untreated papain as 100%. 1, T32Y; 2, T32S; 3, Y89F; 4, T31V; 5, T31S; 6, T32E; 7, wild type; 8, Y89S; 9, T32A; 10, T31Y; 11, T32V; 12, W93A; 13, P30A; 14, T31A/T32A. (B) NMR spectra of the chagasin variant ΔT31–T32. Comparison of ^15^N HSQC spectra recorded at 600 MHz (^1^H) and 298 K, of wild type chagasin (black) with variant ΔT31–T32 (blue), indicating that this variant is properly folded.

Chagasin is highly thermo-stable, retaining its inhibitory activity even after boiling [1,5]. We performed temperature stability assays to examine whether the introduced amino acid substitutions caused detrimental effects on the tertiary structure and to stability of chagasin mutants, as previously reported to occur with cystatin C, another CP inhibitor [15]. No major effect on the inhibitory activity of chagasin or most of the variants was detected by treatment at 70 °C (Fig. 2), indicating that the mutations did not significantly affect the overall structure of the proteins. The T32Y variant had 20% loss of inhibitory activity, suggesting that minor structural/folding alterations had resulted from the mutation introduced. The Y89S and T31Y variants kept on ice were about 40% less active than chagasin, even when tested at large excess over papain, indicating that they had lower affinity for this CP. The variants Y89A and ΔT31-T32 had no inhibitory activity against papain. Analysis of their NMR spectra revealed that the Y89A mutant was partially or completely unfolded (not shown), it was thus excluded from further investigation. However, the ΔT31-T32 variant remained folded (Fig. 2B), suggesting its lack of inhibitory activity against papain was not due to loss of conformation.

### 3.2 Enzyme inhibition properties of chagasin variants

The equilibrium constants for dissociation (*K*_*i*_) of the variants and chagasin from complexes with the *T. cruzi* CP cruzipain and human cathepsin L were determined from continuous assays (Table 2). In order to have an accurate estimation of the amount of active inhibitor present, the preparations were titrated against papain. The *K*_*i*_ value of recombinant His-tagged wild type chagasin with cruzipain and papain were essentially the same as reported for native chagasin purified from *T. cruzi* [1], so we performed all kinetic assays with His-tagged mutants.

The substitution of Y89, which caps the hydrophobic core of chagasin in the 7th β-strand, to either S or F had no impact in the inhibition of cruzipain. In contrast, Y89S was 60-fold less potent than the wild type protein (designated WT) for the inhibition of papain (WT *K*_*i*_ = 5 ± 0.7 pM; Y89S *K*_*i*_ = 316 ± 17 pM), while the *K*_*i*_ of the Y89F variant was unchanged. These results suggest that the bulky hydrophobic side chain of Y89 is important for chagasin’s inhibitory function against papain, while it is unnecessary for the inhibition of cruzipain. The crystal structure of chagasin indicates that residues Y89, D99 and E101 make contact with Loop4 (L4), although such contacts are not observed in the peptidase–chagasin complexes [8–10]. In addition, water molecule-mediated hydrogen bonding between the hydroxyl group of Y89 and G66 (L4) and between Y89, S62 and T32 (L2) were observed, suggesting that Y89 contributes to maintaining the relative orientations of L2 and L4 [9,10]. Although the result with Y89F indicates that the hydrogen bonding is dispensable for the maintenance of chagasin’s tertiary structure, the finding that the Y89A variant, but not Y89S, was unfolded suggests that in the absence of a bulky residue at position 89 the H-bonding mediated by serine becomes important in preventing the collapse of the tertiary structure.

Intriguingly, the W93A mutant inhibited cruzipain in the same range as WT, but displayed 100-fold less affinity for cathepsin L. This suggests that interactions with residues of L6 might be selectively important for chagasin’s inhibition of this host peptidase. Residues R91/P92/W93 (RPW) in L6 form an apparently critical tripartite peptidase recognition motif, as recently described in detail for the interaction with cathepsin L [10]. In the complex, W93 resides within a hydrophobic cluster with the peptidase residues. This causes R91 to lie in extended conformation sandwiched between W93 and F34 (L2), such that R91 hydrogen bonds with N18 of cathepsin L. It seems likely that removal of W93 disturbed the R91–N18 interaction, and that this is important to fix chagasin tightly to cathepsin L.

The functional characterization of a large number of variants bearing substitutions in L2 showed that this domain is generally important for the inhibitory function of chagasin. Interestingly, the relative contribution of the different residues varied with the target enzyme, and the main conclusions that can be drawn from the inhibition assays are as follows.

The mutation P30A caused a sixfold increase in the *K*_*i*_ for cathepsin L and showed little change in affinity for cruzipain. The structure of chagasin revealed that this residue apparently contributes to the conformation of L2 through packing against Y58 of chagasin’s hydrophobic core. Based on this, the mutation to alanine was expected to result in weaker binding to the peptidases due to looser packing of L2 with the more flexible L2 loop incurring a greater entropic penalty in becoming fixed in the complex. However, greater flexibility of L2 in the complex, permitted by the mutation, might make this change rather neutral resulting in only a small change of affinity, as is observed.

The evolutionarily conserved T31 plays a pivotal role in the inhibition of cruzipain, while it is of relatively low importance for the inhibition of cathepsin L (Table 2). In the chagasin–cathepsin L complex, T31 contacts the peptidase active site cysteine via a water mediated hydrogen bond and helps to define the conformation of L6 such that it cannot be attacked by the peptidase [15]. However, mutations of this residue that remove its bulk (T31S), its side chain hydroxyl (T31V) or both (T31A), or that increase its bulk (T31Y) have virtually no effect on chagasin’s binding to cathepsin L. Our suggested explanation for this is that T31 is effectively partially surface exposed and surrounded by ordered water molecules in both determined peptidase complexes. Thus removal of its sidechain bulk could be compensated by additional water molecules, or a bulkier side chain accommodated by displacing them. In contrast, the removal of the side chain in T31A or the introduction of a bulkier chain in T31Y resulted in a 40–100-fold decrease in affinity for cruzipain, indicating that the contacts made by T31 are more critical for this enzyme (Table 2). Given the similarity between the peptidases in this region, these different effects must mean that either there are additional interactions yet to be realised, or that there are significantly different dynamic/entropic properties of the interface in the two complexes.

The conserved T32 also contributes to the binding, but to a lesser extent. Removal of the side chain in T32A, the increase of its length and addition of negative charge in T32E, or the introduction of a bulky side chain in T32Y, had no effect on the affinity for cruzipain (Table 2). In contrast, removal of the hydroxyl group in T32V caused a significant increase in the *K*_*i*_. T32 has well defined roles satisfying intra-molecular hydrogen bonds and defining the conformation of L2. However, our findings that this residue is highly tolerant of substitution suggest that changes in the side chain can be easily accommodated by involving additional water molecules or displacing them. Again, cruzipain is more sensitive to alterations of T32, but of the substitutions tested only removing the hydrogen bonding potential while maintaining the bulk of the side chain (T32V) had a large effect.

The roles of the conserved threonine residues in L2 were further addressed by the double mutation T31A-T32A. This variant displayed 140-fold decrease in affinity for cruzipain and a subtle decrease in the affinity for papain (*K*_*i*_ = 27 ± 1.5 pM). Interestingly, its affinity for cathepsin L was slightly increased (Table 2). We generated a mutant devoid of T31 and T32 in order to evaluate the importance of the length of L2. This variant lacked inhibitory activity against papain and showed a great reduction in potency of inhibition of cathepsin L (∼300-fold) and cruzipain (∼700-fold). This suggests that the length of L2 is generally important for inhibitor function, regardless of the target enzyme.

**Fig. 3:**
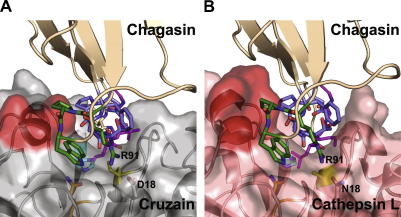
Illustration of the differences in the interaction of chagasin with cysteine peptidases. (A) The chagasin–cruzain interaction model viewed from the “prime” end of the active site coloured as in Fig. 1 with the peptidase “helix 8” region highlighted in red. (B) The same view of the chagasin–cathepsin L complex (PDB:2ndq) illustrating the more extensive interaction surface provided by the peptidase on the “helix 8” lip of the active site.

We also compared the chagasin–cathepsin L complex with a homology model of the chagasin–cruzain complex in an attempt to explain the different behaviour of the variants with the two peptidases (Fig. 3). In the chagasin–cathepin L complex, P92 seems to fix the relative orientations of R91 and W93 in L6 and makes hydrophobic contacts with F145 and L144 of the peptidase (Fig. 3B) [10]. In cruzain, this region adopts a different conformation that protrudes less and is decorated with less hydrophobic residues (Fig. 3A), indicating that cathepsin L has more options to maintain binding when the favourable contribution from T31 is removed. Since these options are not available for cruzain, interaction with T31 might be more crucial in maintaining cruzain–chagasin tight complexes.

In contrast, the W93A mutation had a small effect on binding to cruzipain but a much greater effect on interaction with cathepsin L. Residue R91 of L6 makes a hydrogen bond with cathepsin L N18, while in cruzain, residue 18 is an aspartate which can make an energetically more favourable ionic interaction. A possible explanation for our observation is that cathepsin L, lacking the ionic interaction with D18 as in cruzain, is much more dependent on the supporting role of W93.

Collectively, these data indicate that the structural basis for the function of chagasin as a high-affinity inhibitor of papain-like CPs varies according to the target enzyme, with main contributions of T31 located on L2 for the inhibition of the endogenous CP of the parasite and of W93 located on L6 for the inhibition of host cathepsin L. Structural analyses of the complexes of chagasin with additional peptidases will bring further understanding of the differences in its mode of inhibition of target peptidases.

## Figures and Tables

**Table 1:** Site-directed mutagenesis of chagasin

Mutation	Construct	Oligonucleotide	Sequence
W93A	pGL1199	OL1695	5′-TTTACATGCGCCCG**GC**GACAGGAGCTTCGC-3′
		OL1696	5′-GCGAAGCTCCTGTC**GC**CGGGCGCATGTAAA-3′
			
Y89A	pGL1118	CHAGY1	5′-ATTCTCACT**GC**CATGCGCCCG-3′
		CHAGY2	5′-CGGGCGCATG**GC**AGTGAGATT-3′
			
Y89S	pGL1120	CHAGY3	5′-ATTCTCACTT**C**CATGCGCCCG-3′
		CHAGY4	5′-CGGGCGCATG**G**AAGTGAGATT-3′
			
Y89F	pGL1119	CHAGY5	5′-ATTCTCACTT**T**CATGCGCCCG-3′
		CHAGY6	5′-CGGGCGCATG**A**AAGTGAGATT-3′
			
P30A	pGL1194	OL1679	5′-GCTTCCCAGCAAT**G**CCACCACCGGGTTC-3′
		OL1680	5′-GAACCCGGTGGTGG**C**ATTGCTGGGAAGC-3′
			
T31A	pGL1209	OL1681	5′-CCAGCAATCCC**G**CCACCGGGTTCG-3′
		OL1682	5′-CGAACCCGGTGG**C**GGGATTGCTGG-3′
			
T31S	pGL1299	OL1851	5′-CCAGCAATCCC**TCC**ACCGGGTTCG-3′
		OL1852	5′-CGAACCCGGT**GGA**GGGATTGCTGG-3′
			
T31V	pGL1298	OL1849	5′-CCAGCAATCCC**GTC**ACCGGGTTCG-3′
		OL1850	5′-CGAACCCGGT**GAC**GGGATTGCTGG-3′
			
T31Y	pGL1210	OL1683	5′-CTTCCCAGCAATCCC**TA**CACCGGGTTCGCGTG-3′
		OL1684	5′CACGCGAACCCGGTG**TA**GGGATTGCTGGGAAG-3′
			
T32A	pGL1211	OL1685	5′-GCAATCCCACC**G**CCGGGTTCGCGT-3′
		OL1686	5′-ACGCGAACCCGG**C**GGTGGGATTGC-3′
			
T32S	pGL1195	OL1687	5′-AGCAATCCCACC**T**CCGGGTTCGCGTG-3′
		OL1688	5′-CACGCGAACCCGG**A**GGTGGGATTGCT-3′
			
T32E	pGL1197	OL1691	5′CCCAGCAATCCCACC**GAG**GGGTTCGCGTGGTATTTT-3′
		OL1692	5′AAAATACCACGCGAACCC**CTC**GGTGGGATTGCTGGG-3′
			
T32Y	pGL1198	OL1693	5′-CCAGCAATCCCACC**TA**CGGGTTCGCGTGGT-3′
		OL1694	5′-ACCACGCGAACCCG**TA**GGTGGGATTGCTGG-3′
			
T32V	pGL1196	OL1689	5′-CAGCAATCCCACC**GT**CGGGTTCGCGTGG-3′
		OL1690	5′-CCACGCGAACCCG**AC**GGTGGGATTGCTG-3′
			
T31A/T32A	pGL1258	OL1776	5′-CCCAGCAATCCC**G**CC**G**CCGGGTTCGCGTGG-3′
		OL1777	5′-CCACGCGAACCCGG**C**GG**C**GGGATTGCTGGG-3′
			
ΔT31–T32	pGL1259	OL1778	5′-CAGCTTCCCAGCAATCCCGGGTTCGCGTGGTAT-3′
		OL1779	5′-ATACCACGCGAACCCGGGATTGCTGGGAAGCTG-3′

The pairs of oligonucleotides used to perform site-directed mutagenesis are listed with the respective amino acid substitution. The targeted mutations are marked in bold. *T. cruzi* Dm28c chagasin gene was used as a template in PCR reactions. The mutations were confirmed by DNA sequencing.

**Table 2:** *K*_*i*_ values for chagasin against *T. cruzi* cruzipain and human cathepsin L

Substitution	Region	Cruzipain	Cathepsin L
*K*_*i*_*(pM)*			
Chagasin	–	7.6 ± 0.67	7.3 ± 1.2
			
W93A	L6	31 ± 3.5	825 ± 79
Y89S	β7-strand	25 ± 3.1	ND
Y89F	β7-strand	20 ± 0.8	ND
			
P30A	L2	20 ± 1.7	45 ± 7.3
T31A	L2	317 ± 26	3.9 ± 0.7
T31V	L2	70 ± 4.3	9.7 ± 1.1
T31S	L2	11 ± 0.6	4.9 ± 0.6
T31Y	L2	747 ± 44	6.8 ± 0.9
T32A	L2	16 ± 1.1	14 ± 1.8
T32S	L2	20 ± 1.9	32 ± 2.2
T32E	L2	11 ± 2.0	ND
T32Y	L2	16 ± 0.7	2.7 ± 0.2
T32V	L2	193 ± 13	20 ± 0.8
			
T31A/T32A	L2	1065 ± 116	1.8 ± 0.4
ΔT31–T32	L2	5868 ± 29	2200 ± 17

Equilibrium constants for dissociation (*K*_*i*_) of complexes between cysteine peptidases and chagasin variants. The results are represented as mean values from three independent *K*_*i*_ measurements. The enzymes were assayed for inhibition as described in methods. N.D., not determined. The values are mean of three independent *K*_*i*_ determinations ± S.E. The conditions of hydrolysis: 50 mM Na_2_HPO_4_, 100 mM NaCl, 5 mM EDTA, pH 6.5, 5% DMSO, 2.5 mM DTT, at 28 °C.
